# Supplementary material for: The causal role of circulating inflammatory markers in osteoporosis: a bidirectional Mendelian randomized study
Source: Front Immunol. 2024 Jul 18;15:1412298. doi: 10.3389/fimmu.2024.1412298 (PMC11291241; doi:10.3389/fimmu.2024.1412298)
Supplement: Supplementary file 1 [file DataSheet_1.zip › supplementary material/CIM-OSTEOPOROSIS /GCST90274847.csv_leave_one_out.pdf]

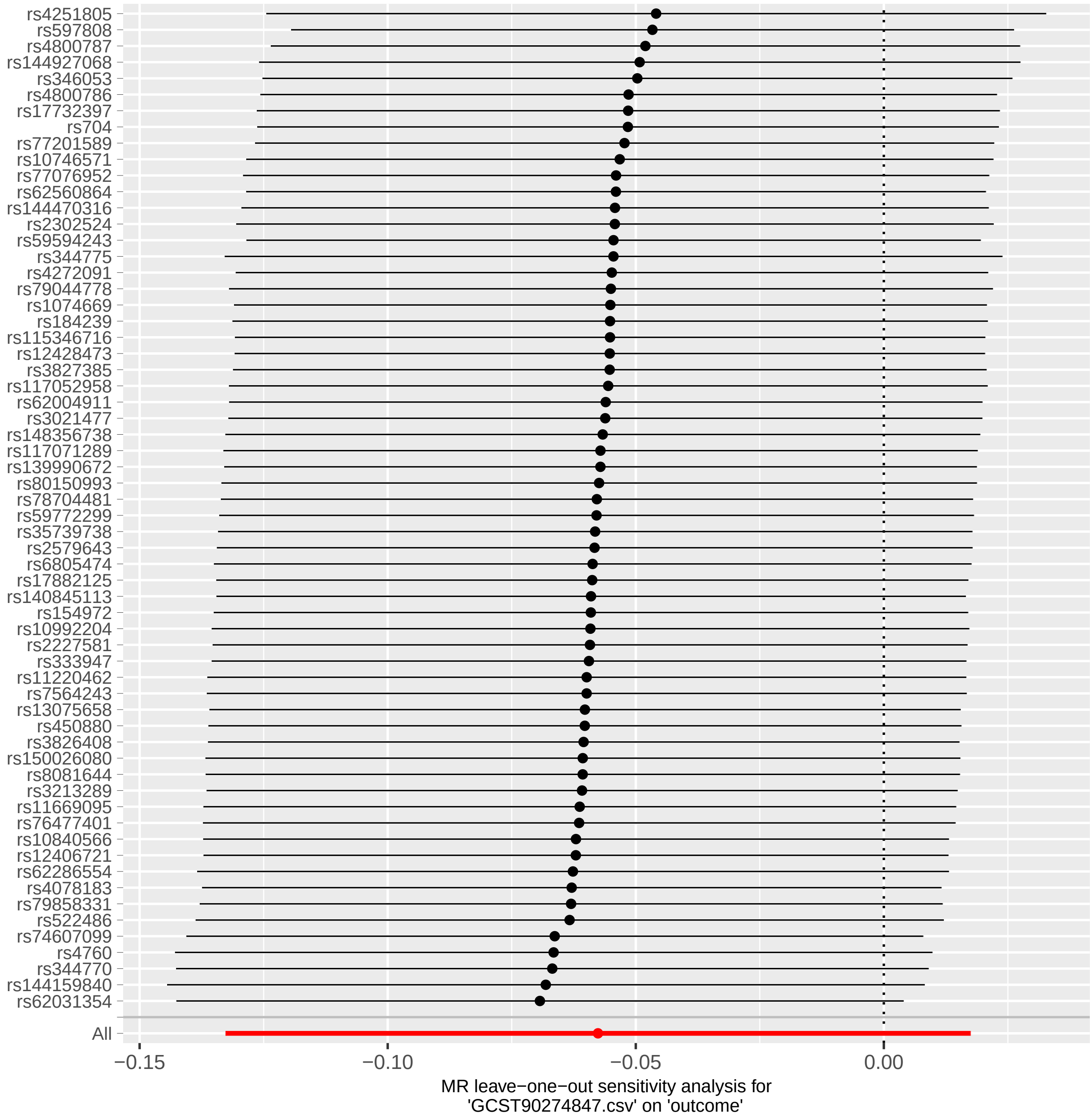
rs4251805
rs597808
rs4800787
rs144927068
rs346053
rs4800786
rs17732397
rs704
rs77201589
rs10746571
rs77076952
rs62560864
rs144470316
rs2302524
rs59594243
rs344775
rs4272091
rs79044778
rs1074669
rs184239
rs115346716
rs12428473
rs3827385
rs117052958
rs62004911
rs3021477
rs148356738
rs117071289
rs139990672
rs80150993
rs78704481
rs59772299
rs35739738
rs2579643
rs6805474
rs17882125
rs140845113
rs154972
rs10992204
rs2227581
rs333947
rs11220462
rs7564243
rs13075658
rs450880
rs3826408
rs150026080
rs8081644
rs3213289
rs11669095
rs76477401
rs10840566
rs12406721
rs62286554
rs4078183
rs79858331
rs522486
rs74607099
rs4760
rs344770
rs144159840
rs62031354
All
−0.15
−0.10
−0.05
0.00
MR leave−one−out sensitivity analysis for
'GCST90274847.csv' on 'outcome'
